# Prehabilitation models in patients undergoing radical prostatectomy: A scoping review

**DOI:** 10.1371/journal.pone.0356745

**Published:** 2026-08-27

**Authors:** Yilei Li, Qin Chen, Hong Chen, Yan Chen, Xiangru Huang, Haixia Li

**Affiliations:** 1 School of Nursing, Ningxia Medical University, Yinchuan, Ningxia, China; 2 People’s Hospital of Ningxia Hui Autonomous Region, Yinchuan, Ningxia, China; Jan Biziel University Hospital No 2 in Bydgoszcz: Szpital Uniwersytecki Nr 2 im dr Jana Biziela w Bydgoszczy, POLAND

## Abstract

Prehabilitation, defined as the preoperative optimisation of physical, nutritional, and psychological status, has emerged as a promising strategy to improve postoperative recovery in surgical oncology. However, its application in patients undergoing radical prostatectomy (RP) remains poorly characterised. This scoping review aimed to systematically map existing evidence on prehabilitation models for patients scheduled for RP, with particular focus on delivery formats, intervention components, and outcome measures. Following the Arksey and O’Malley framework and reported in accordance with the Preferred Reporting Items for Systematic Reviews and Meta-Analyses extension for Scoping Reviews (PRISMA-ScR) guidelines, a comprehensive search of nine electronic databases was conducted from inception to 1 December 2025. Thirteen studies from eight countries were included, comprising randomised controlled trials (RCTs), quasi-experimental studies, and prospective cohort studies. Three distinct delivery formats were identified: digital remote interventions via mobile applications (n = 4), non-digital remote interventions via booklets and telephone follow-up (n = 4), and in-person hospital-based programmes (n = 5). Pelvic floor muscle training (PFMT) was the most consistently implemented component (n = 11), complemented by aerobic exercise, resistance training, nutritional support, psychological interventions, and health education. Outcome measures spanned six domains: urinary continence and sexual function, physical function, perioperative clinical outcomes, psychological status, health-related quality of life (HRQoL), and nutritional status. Considerable heterogeneity was observed across intervention content and outcome assessment instruments. Prehabilitation appears feasible and acceptable across diverse delivery formats; however, the current evidence base is constrained by methodological heterogeneity and inconsistent outcome measurement. High-quality RCTs are warranted to establish standardised multimodal prehabilitation protocols and consensus-based core outcome sets for this population.

## Introduction

Prostate cancer (PCa) is among the most prevalent malignancies affecting men globally, representing a substantial and growing public health burden [1]. Radical prostatectomy (RP) remains a primary curative treatment for clinically localised PCa [2]; however, the procedure is associated with a range of adverse postoperative sequelae. Postoperative urinary incontinence represents the most frequently reported complication, with reported rates of 21.3% following robot-assisted RP and 20.2% following open retropubic RP [3]. Beyond its clinical significance, urinary incontinence substantially impairs patients’ health-related quality of life and imposes considerable psychological and socioeconomic burdens on both patients and healthcare systems [4].

Prehabilitation has emerged as a key component of enhanced recovery after surgery (ERAS) protocols, referring to a multimodal preoperative intervention strategy encompassing exercise training, nutritional optimisation, psychological support, and health behaviour education [5,6]. The theoretical rationale underpinning prehabilitation posits that improving patients’ functional reserve and physiological resilience prior to surgical intervention may attenuate treatment-related deconditioning and accelerate postoperative recovery. In recognition of this potential, the Chinese Expert Consensus on Integrated Perioperative Rehabilitation for Radical Prostatectomy (2024 Edition) has formally incorporated prehabilitation into standardised clinical care pathways, identifying the preoperative period as a critical window of opportunity for targeted intervention [7]. Despite this growing clinical endorsement, substantial unmet need for structured professional guidance among patients persists, and the translation of prehabilitation into routine clinical practice remains limited.

Although several studies have examined prehabilitation within this population, the existing evidence is characterised by considerable heterogeneity in intervention content, delivery formats, and outcome measures. Notably, no scoping review has yet comprehensively mapped the distribution and characteristics of this body of evidence. While previous systematic and scoping reviews have explored prehabilitation across various cancer populations [8,9], these investigations have not specifically addressed the unique clinical and rehabilitative context of RP. A PCa-specific scoping review is warranted for several important reasons. First, RP presents distinct and well-defined recovery challenges—most notably urinary incontinence, erectile dysfunction, and psychological distress—that necessitate tailored prehabilitation strategies rather than the application of generic cancer prehabilitation frameworks. Second, the increasing adoption of minimally invasive surgical approaches and the progressive integration of ERAS protocols have created new opportunities for preoperative optimisation that require dedicated investigation. Third, the PCa patient population is predominantly older, presenting specific considerations that may influence the design, acceptability, and feasibility of digital and remote intervention delivery.

To address this evidence gap, this scoping review aimed to systematically map prehabilitation interventions for patients undergoing RP, characterising their delivery formats, intervention components, and outcome measures, thereby providing a comprehensive evidence base to inform future research priorities and guide clinical practice development.

## Methods

### Study design

This scoping review was conducted in accordance with the methodological framework proposed by Peters et al [10]. and reported following the Preferred Reporting Items for Systematic Reviews and Meta-Analyses extension for Scoping Reviews (PRISMA-ScR) guidelines [11]. The review protocol was retrospectively registered on the Open Science Framework (OSF; https://doi.org/10.17605/OSF.IO/TG235).

### Identifying the research questions

This scoping review was guided by three a priori research questions, developed in accordance with the study objectives and structured using the Population, Concept, and Context (PCC) framework:

RQ1: What delivery formats are used in prehabilitation interventions for patients scheduled for radical prostatectomy?
RQ2: What components are included in these prehabilitation interventions?
RQ3: What outcome measures are reported in studies examining prehabilitation for patients scheduled for radical prostatectomy?

### Inclusion and exclusion criteria

Eligibility criteria were developed a priori using the PCC framework to ensure systematic and transparent study selection.

Population: Adult male patients diagnosed with prostate cancer (PCa) and scheduled to undergo radical prostatectomy (RP) (including open, laparoscopic, or robot-assisted approaches) were eligible for inclusion. No restrictions were imposed on age, disease stage, or comorbidity profile.

Concept: Any form of preoperative prehabilitation intervention was eligible, including unimodal interventions (e.g., exercise training, nutritional optimisation, psychological support, or pelvic floor muscle training [PFMT]) and multimodal interventions combining two or more components. To be eligible, interventions must have been initiated prior to the scheduled surgical date. Studies in which the primary intervention was delivered exclusively in the postoperative period were excluded; however, studies that reported postoperative outcomes following a preoperative prehabilitation were retained for inclusion.

Context: All clinical practice settings were eligible, including hospital- based, community-based, home-based, and telemedicine environments. No restrictions were applied to geographic region, healthcare system type, cultural context, or sample size.

Exclusion criteria: Studies were excluded if they met any of the following criteria: (1) published in languages other than English or Chinese; (2) full text was unavailable; (3) secondary literature, including systematic reviews, scoping reviews, narrative reviews, meta-analyses, study protocols, conference abstracts, editorials, or commentaries; (4) duplicate publications. The restriction to English- and Chinese-language publications is acknowledged as a potential source of language bias; this limitation is discussed further in the Limitations section of this review.

### Search strategy

A systematic literature search was conducted across nine databases and platforms: PubMed, Web of Science, Embase, Cochrane Library, Scopus, China National Knowledge Infrastructure (CNKI), Wanfang Medical Database, China Biomedical Literature Database (SinoMed), and VIP Chinese Journal Service Platform. The search strategy combined Medical Subject Headings (MeSH) terms and free-text keywords. The search timeframe spanned from database inception to 1 December 2025, with no language restrictions applied at the search stage. For Chinese databases, corresponding Chinese-language search terms were used, covering prostate cancer and related diagnoses, and prehabilitation and related rehabilitation concepts. The complete search strategies for all databases are provided in Supplementary File S6 to ensure transparency and reproducibility of the search process. The PubMed search strategy is presented in Table 1 as an illustrative example.

**Table 1:** PubMed search strategy.

Step	Search Strategy
#1	“Prostatic Neoplasms”[Mesh]
#2	“prostat* neoplasm*”[Title/Abstract] OR “prostat* cancer*”[Title/Abstract] OR “cancer of prostat*”[Title/Abstract] OR “cancer of the prostat*”[Title/Abstract]
#3	#1 OR #2
#4	“Prostatectomy”[Mesh]
#5	“prostatectom*”[Title/Abstract] OR “radical prostatectom*”[Title/Abstract] OR “robot* prostatectom*”[Title/Abstract] OR “laparoscop* prostatectom*”[Title/Abstract]
#6	#4 OR #5
#7	“Preoperative Exercise”[Mesh] OR “Preoperative Care”[Mesh] OR “Exercise Therapy”[Mesh] OR “Physical Conditioning, Human”[Mesh]
#8	“prehab*”[Title/Abstract] OR “preoperat* exercis*”[Title/Abstract] OR “preoperat* rehab*”[Title/Abstract] OR “preoperat* train*”[Title/Abstract] OR “preoperat* optim*”[Title/Abstract] OR “preoperat* interven*”[Title/Abstract] OR “preoperat* program*”[Title/Abstract] OR “exercise train*”[Title/Abstract] OR “physical train*”[Title/Abstract] OR “pelvic floor muscle train*”[Title/Abstract] OR “surgical prehab*”[Title/Abstract] OR “multimodal prehab*”[Title/Abstract]
#9	#7 OR #8
#10	#3 AND #6 AND #9

### Study selection

Retrieved records were imported into NoteExpress(version 4.2.0; Aegean Sea Software, China) reference management software for deduplication and systematic screening. Two trained researchers independently screened all titles and abstracts against the predetermined eligibility criteria, followed by independent full-text review of potentially eligible articles. Disagreements arising at any stage of the screening process were resolved through structured discussion, where consensus could not be reached, a third researcher consulted for adjudication.

### Data extraction

Data were extracted from each included study using a standardised data extraction form developed and pilot-tested by the research team prior to full implementation. The following information was systematically extracted from each eligible study: study design; sample size; intervention duration; delivery format; intervention content (components and modalities); and outcome measures. Data extraction was performed independently by two researchers, with discrepancies resolved through discussion or, where necessary, consultation with a third researcher.

### Data synthesis

A structured narrative synthesis was conducted in three sequential stages. First, included studies were independently grouped by delivery format and intervention components by two researchers; emergent categories were subsequently refined through iterative team consensus. Second, a standardised coding framework was applied, with two researchers independently coding intervention components and delivery formats; inter-rater calibration was achieved through pilot coding of a randomly selected sample of two included studies prior to full-scale extraction. Third, disagreements between coders were resolved through consensus meetings, with any unresolved discrepancies adjudicated by a third researcher. The synthesis was explicitly oriented towards mapping the landscape of prehabilitation models rather than evaluating intervention effectiveness. Outcome domains were inductively derived from the extracted data, with proposed categories independently generated and refined through consensus discussion. Findings are presented narratively and are supplemented by summary tables and figures to facilitate interpretation.

### Ethical approval

Ethical approval was not required for this scoping review, as the study involved exclusively the analysis of previously published literature and did not include any primary data collection involving human participants.

## Results

### Study selection

**Fig 1:**
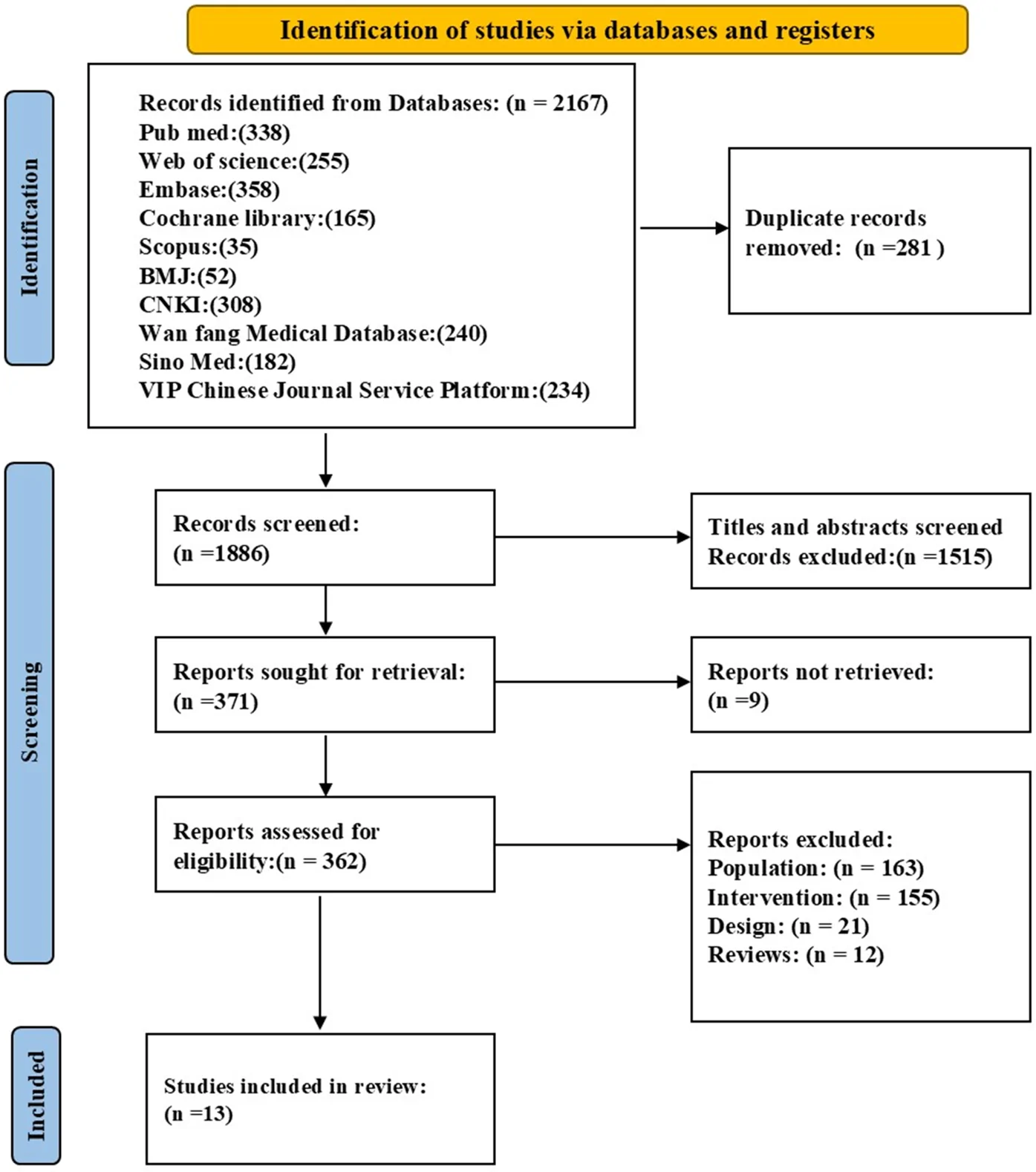
PRISMA-ScR flow diagram illustrating the study selection process. Records were identified through systematic searching of nine electronic databases. Following removal of duplicates and sequential screening, 13 studies were ultimately included in this scoping review.

The initial database search yielded 2,167 records. Following removal of duplicates, sequential title and abstract screening, and independent full-text review, 13 studies were ultimately included in this scoping review [12–24]. The complete literature screening process is presented in Fig 1.

### Characteristics of included studies

**Fig 2:**
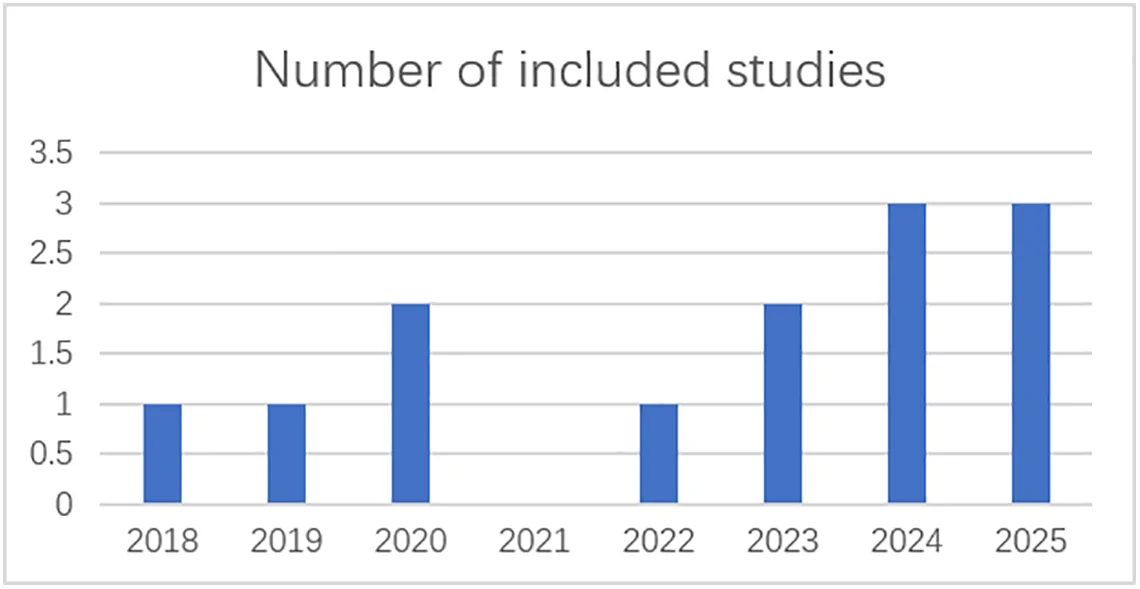
Publication timeline of the included studies (n = 13). The X-axis represents the publication year, and the Y-axis represents the number of studies published in each year.

**Fig 3:**
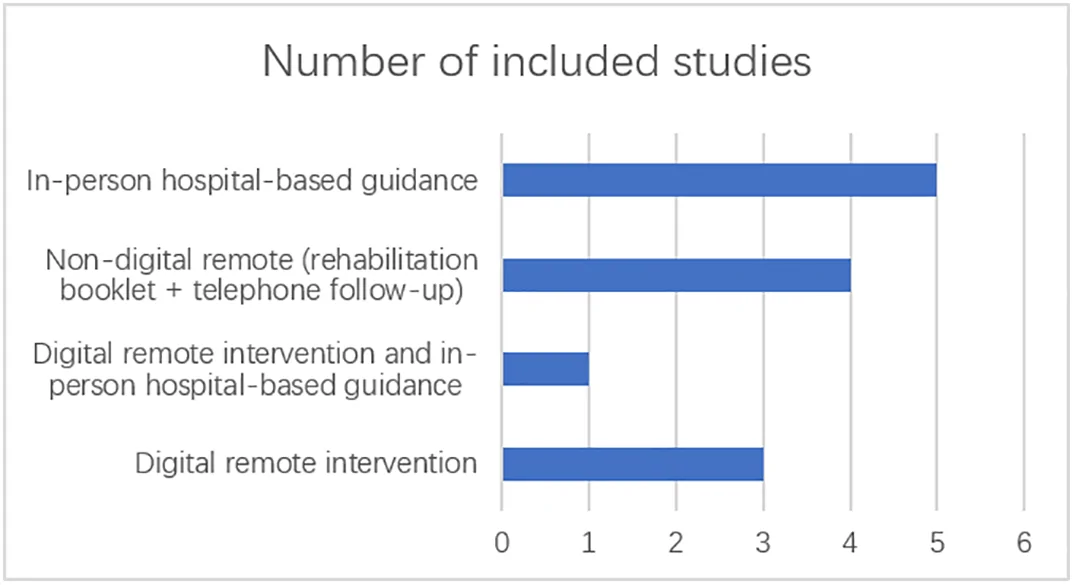
Distribution of intervention delivery models across the included studies (n = 13). The table presents the number of included studies for each intervention delivery model. Delivery models were categorized as: (1) in-person hospital-based guidance, (2) non-digital remote (rehabilitation booklet + telephone follow-up), (3) digital remote intervention, and (4) digital remote intervention and in-person hospital-based guidance.

**Fig 4:**
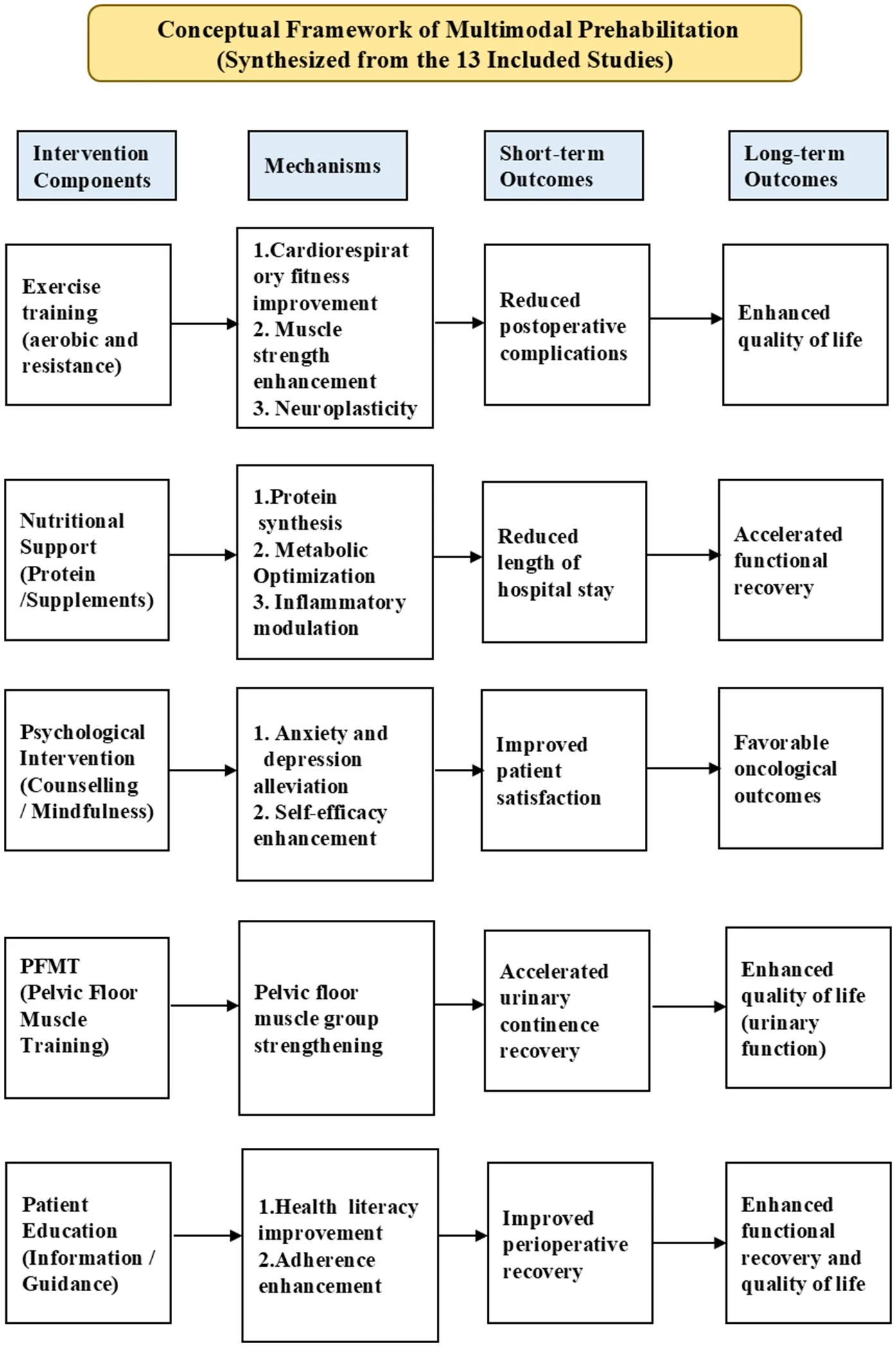
Conceptual framework of multimodal prehabilitation for patients undergoing radical prostatectomy, synthesized from the included studies (n = 13).

The 13 included studies were published between 2018 and 2025 (Tables 2–6 and Figs 2–4). The majority of studies (n = 8) originated from European countries (France, Germany, and Denmark), with three from China, and one each from Australia, Canada, and Brazil. Study designs comprised six RCTs[17–22]and seven quasi-experimental or prospective studies [12–16,23,24] Sample sizes ranged from 31 to 544 participants. Intervention durations varied considerably, from a single-day multidisciplinary workshop [16] to six weeks of structured programming [17]; several studies continued interventions until the date of surgery [12,21,24], while two studies did not report intervention duration [14,20].

**Table 2:** Basic characteristics of the included studies (n = 13).

Included Study	Country	Year of Publication	Study Design	Sample Size (Intervention/Control, n)	Intervention Duration
Alberto Martini et al. [12]	Germany	2024	Quasi-experimental study	60/62	From diagnosis to 6 weeks post-surgery
Alessandro Uleri et al. [13]	France	2025	Prospective study	156/177	2 weeks pre-surgery
Bogdan Adrian Buhas et al. [14]	France	2025	Prospective study	146/204	Not reported
Guillaume Ploussard et al. [15]	France	2020	Quasi-experimental study	194/350	1 week pre-surgery
Razvan-George Rahota et al. [16]	France	2022	Quasi-experimental study	165/138	1 day pre-surgery
Favil Singh et al. [17]	Australia	2023	RCT	20/21	6 weeks pre-surgery
Malene Blumenau Pedersen et al. [18]	Denmark	2024	RCT	20/20	4 weeks pre-surgery
Santa Mina D et al. [19]	Canada	2018	RCT	44/43	4 weeks pre-surgery
de Lira GHS et al. [20]	Brazil	2019	RCT	16/15	Not reported
Zhang Xinhong et al. [21]	China	2024	Quasi-experimental study	60/60	From diagnosis to pre-surgery
Huang Yuhan et al. [22]	China	2020	RCT	30/30	From admission to 1 day pre-surgery (approximately 7–10 days)
Nie Ting et al. [23]	China	2023	Quasi-experimental study	35/35	2 weeks pre-surgery
Wang Fengying et al. [24]	China	2025	Quasi-experimental study	41/41	From diagnosis to pre-surgery

**Table 3:** Intervention formats and content of the included studies (n = 13).

Included Study	Intervention Format	Intervention Content
Alberto Martini et al. [12]	Digital remote intervention	Exercise: moderate-intensity aerobic training, walking plans, and cardiorespiratory fitness exercises; Pelvic floor muscle training: preoperative pelvic floor exercise guidance via the Betty.care application (videos and checklists); Nutritional support: weight management counselling and oral nutritional supplementation guidance; Health education: educational materials (videos and articles) on preoperative status optimisation, key care arrangements includingconsultation, admission, discharge, and postoperative follow-up.
Alessandro Uleri et al. [13]	Digital remote intervention	Pelvic floor muscle training: pelvic floor exercise videos, articles, and daily exercise checklists via the Betty.care application; Health education: questionnaires, checklists, video information onurinary and erectile function risks, bladder catheter management guidance, and information on potential postoperative adverse events; key care arrangements including consultation, admission, discharge, and postoperative follow-up.
Bogdan Adrian Buhas et al. [14]	Digital remote intervention and in-person hospital-based guidance	Digital remote: Betty.care application providing pelvic floor exercise videos, daily reminders, task lists, exercise and nutritional recommendations, and educational courses; In-person: multidisciplinary team teaching covering pelvic floor muscle exercises, exercise planning, breathing exercises, urinary catheter care, nutritional optimisation, and analgesia education.
Guillaume Ploussard et al. [15]	Non-digital remote (rehabilitation booklet + telephone follow-up)	Exercise: walking plans, aerobic training, and breathing exercises;Pelvic floor muscle training: pelvic floor muscle exercises; Nutritional support: nutritional assessment, oral nutritional supplementation, dietary supplements, and weight management counselling.
Razvan-George Rahota et al. [16]	In-person hospital-based guidance	Exercise: walking plans, aerobic training, and breathing exercises;Pelvic floor muscle training: pelvic floor muscle exercises; Nutritional support: nutritional assessment, oral nutritional supplementation, dietary supplements, and weight management counselling.
Favil Singh et al. [17]	Non-digital remote (rehabilitation booklet + telephone follow-up)	Exercise: resistance training targeting major muscle groups (leg press, leg extension, leg curl, chest press, seated row, lat pulldown, triceps extension, bicep curl, and calf raises); core stability training (planks, Swiss ball reverse bridges, and side planks); aerobic training (treadmill walking/jogging, stationary cycling, or rowing).
Malene Blumenau Pedersen et al. [18]	Digital remote intervention	Exercise: unsupervised individualised aerobic exercise (moderate intensity) and resistance training; Pelvic floor muscle training: physiotherapist-led assessment of pelvic floor muscle function, education on pelvic anatomy and muscle function, and a personalised pelvic floor exercise plan guidedby real-time ultrasound; Nutritional support: nutritional status and malnutrition risk screening (NRS-2002); dietary advice via application; nutritional supplementation one week preoperatively for patients in accordance with ESPEN guidelines; Psychological support: systematic screening for anxiety and depression (HADS); counselling with coping strategies for patients at risk (HADS > 8); Sexual counselling: one video consultation with a clinical sexologist (based on IIEF-5 responses), covering communication strategies, realistic expectations, erectile dysfunction management, and use of assistive devices.
Santa Mina D et al. [19]	Non-digital remote (rehabilitation booklet + telephone follow-up)	Exercise: upper limb resistance training (resistance band and dumbbell exercises); lower limb aerobic training (brisk walking, stair climbing, and cycling); Pelvic floor muscle training: pelvic floor muscle exercises.
de Lira GHS et al. [20]	In-person hospital-based guidance	Pelvic floor muscle training: pelvic floor muscle exercises with electromyography-based biofeedback.
Zhang Xinhong et al. [21]	In-person hospital-based guidance	Exercise: abdominal breathing exercises; Pelvic floor muscle training: pelvic floor muscle exercises; Nutritional support: nutritional risk screening (NRS-2002); oral nutritional supplementation and enteral nutrition for patients withNRS-2002 ≥ 3; dietary protein recommendations (80–120 g/day)and preoperative whey protein supplementation for patients withNRS-2002 < 3; Psychological intervention: mindfulness-based intervention, music therapy combined with progressive muscle relaxation; Lifestyle modification: smoking and alcohol cessation, increased fluid intake,and increased intake of lycopene and selenium.
Huang Yuhan et al. [22]	In-person hospital-based guidance	Exercise: upper limb resistance training (resistance band and dumbbell exercises); lower limb aerobic training (brisk walking, stair climbing, and cycling); breathing exercises (effective coughing and pursed-lip breathing); Nutritional support: nutritional risk screening (NRS-2002); oral and enteral nutritional support with parenteral nutrition if necessary for patients with NRS-2002 ≥ 3;dietary counselling (smoking and alcohol cessation, blood glucosecontrol, balanced diet, high-quality protein and dietary fibre intake) for patients with NRS-2002 < 3; Health education: preoperative cancer prehabilitation education delivered by the attending physician and responsible nurse.
Nie Ting et al. [23]	Non-digital remote (rehabilitation booklet + telephone follow-up)	Exercise: effective coughing training, abdominal breathing exercises, and ankle pump exercises; Pelvic floor muscle training: pelvic floor muscle exercises; Nutritional support: nutritional risk screening; oral nutritional supplementation, enteral nutrition support, and parenteral nutritionas indicated; Psychological intervention: psychological status assessment; progressive muscle relaxation, meditation, mindfulness-based intervention, acceptance and commitment therapy, and cognitive behavioural restructuring training.
Wang Fengying et al. [24]	In-person hospital-based guidance	Exercise: daily 30-minute walking; Pelvic floor muscle training: pelvic floor muscle exercises; Nutritional support: nutritional risk screening; high-protein dietary recommendations for patients requiring nutritional supplementation; Psychological intervention: sleep restriction therapy, relaxation training (4-7-8 breathing technique), mindfulness-based stress reduction training, and psychological status assessment with referral for counselling when necessary; Health education: preoperative pain management education.

**Table 4:** Evidence map of intervention components included studies (n = 13).

Included Study	Exercise	Nutrition	Psychological	PFMT	Education	Other
Alberto Martini et al., 2024 [12]	Y	Y	N	Y	Y	N
Alessandro Uleri et al., 2025 [13]	N	N	N	Y	Y	N
Bogdan Adrian Buhas et al., 2025 [14]	Y	Y	N	Y	Y	N
Guillaume Ploussard et al., 2020 [15]	Y	Y	N	Y	N	N
Razvan-George Rahota et al., 2022 [16]	Y	Y	N	Y	N	N
Favil Singh et al., 2023 [17]	Y	N	N	N	N	N
Malene Blumenau Pedersen et al., 2024 [18]	Y	Y	Y	Y	Y	Y(Sexual counselling)
Santa Mina D et al., 2018 [19]	Y	N	N	Y	N	N
de Lira GHS et al., 2019 [20]	N	N	N	Y	N	N
Zhang Xinhong et al., 2024 [21]	Y	Y	Y	Y	Y	Y(Dietary counselling)
Huang Yuhan et al., 2020 [22]	Y	Y	N	Y	N	N
Nie Ting et al., 2023 [23]	Y	Y	Y	Y	N	N
Wang Fengying et al., 2025 [24]	Y	Y	Y	Y	Y	N
Total (n)	11	9	4	12	6	2

Each row represents one included study, and each column represents a specific intervention component. Y = Yes, component included; N = No, component not included.

**Table 5:** Outcome measures of the included studies (n = 13).

Included Study	Outcome Measures
Alberto Martini et al. [12]	Urinary continence recovery (defined as use of ≤1 pad/day at 6 weeks post-RP);length of hospital stay; same-day discharge rate; readmission rate; days alive and out of hospital within 30 days post-surgery; perioperative complications (Clavien-Dindo classification).
Alessandro Uleri et al. [13]	Urinary continence recovery (defined as use of 0–1 pad/day at 6 weeks post-RP); erectile function at 6 months; perioperative complications (Clavien-Dindo classification); unplanned hospital visits.
Bogdan Adrian Buhas et al. [14]	Urinary continence recovery (defined as use of 0–1 pad/day at 6 weeks post-RP); complication rate; 24-hour discharge rate; unplanned hospital visits; readmission rate; patient satisfaction.
Guillaume Ploussard et al. [15]	Length of hospital stay (mean length of stay; prolonged stay defined as >2 days); intraoperative blood loss; operative time; readmission rate; 90-day overall survival; total hospitalisation costs.
Razvan-George Rahota et al. [16]	Urinary continence recovery (defined as no pad use at 1 and 6 months post-RP); surgical parameters (operative date and duration, intraoperative blood loss); length of hospital stay; 90-day readmission rate (emergency department and/or urology readmissions); unplanned hospital visits (readmissions and unscheduled urology visits); complication rate (Clavien-Dindo classification).
Favil Singh et al. [17]	Physical function: dynamic muscle strength (chest press and leg press), repeated chair stand, stair climbing, 400 m walk, and 6 m fast and backward walk; body composition; urinary incontinence severity (24-hour pad test); fatigue (EORTC QLQ-C30 fatigue subscale); quality of life (EORTC QLQ-C30 global health scale); length of hospital stay.
Malene Blumenau Pedersen et al. [18]	Cardiorespiratory fitness (6-Minute Walk Test [6MWT]); lower limb functional strength (30-Second Sit-to-Stand Test [30STS]); upper limb strength (handgrip dynamometry); self-reported physical activity level; psychological status (Hospital Anxiety and Depression Scale [HADS]); health-related quality of life (12-Item Short-Form Health Survey [SF-12]); erectile function (5-item International Index of Erectile Function [IIEF-5]); urinary incontinence severity (24-hour pad test); nutritional status (Nutritional Risk Screening [NRS-2002]).
Santa Mina D et al. [19]	Cardiorespiratory fitness (6MWT); upper limb strength (handgrip dynamometry); quality of life (Functional Assessment of Cancer Therapy-Prostate [FACT-P]); psychological status (State-Trait Anxiety Inventory [STAI]; Center for Epidemiologic Studies Depression Scale [CES-D]); urinary incontinence severity (International Consultation on Incontinence Questionnaire-Urinary Incontinence Short Form [ICIQ-UI SF]; 24-hour pad test); physical activity level (Godin Leisure-Time Exercise Questionnaire [GLTEQ]).
de Lira GHS et al. [20]	Urinary incontinence severity (ICIQ-UI SF); erectile function (IIEF-5); pelvic floor muscle function: electromyographic activity (mean and maximum values during resting state and rapid sustained contractions, recorded using the Miotool Uro device [Miotec®, Porto Alegre, Brazil]).
Zhang Xinhong et al. [21]	Cardiorespiratory fitness (6MWT); psychological status (Hospital Anxiety and Depression Scale [HADS]); postoperative recovery: time to first ambulation, time to first flatus, discharge readiness score (Readiness for Hospital Discharge Scale [RHDS]); overall complication rate; length of hospital stay.
Huang Yuhan et al. [22]	Cardiorespiratory fitness (6MWT); psychological status (Self-Rating Depression Scale [SDS]; Self-Rating Anxiety Scale [SAS]); quality of life (European Organisation for Research and Treatment of Cancer Quality of Life Questionnaire-Prostate [EORTC QLQ-PR25]); postoperative recovery: time to first ambulation, time to urinary catheter removal, time to drain removal; postoperative length of hospital stay; hospitalisation costs; overall complication rate.
Nie Ting et al. [23]	Postoperative recovery: time to first flatus, time to first ambulation, incidence of nausea and vomiting, postoperative pain severity (Visual Analogue Scale [VAS] at 0.5, 6, 24, and 48 h post-surgery); postoperative length of hospital stay; urinary continence (International Continence Society [ICS] criteria at 3 months post-discharge); sexual function (IIEF-5; Sexual Life Quality Questionnaire [SLQQ-QOL]); psychological status: disease uncertainty (Mishel Uncertainty in Illness Scale [MUIS], Chinese version); fear of progression (Fear of Progression Questionnaire-Short Form [FoP-Q-SF], Chinese version); quality of life (Functional Assessment of Cancer Therapy-General [FACT-G]).
Wang Fengying et al. [24]	Urinary function: voided volume, post-void residual volume, maximum bladder capacity, maximum urinary flow rate (urodynamic evaluation); sleep quality (Insomnia Severity Index [ISI]).

**Table 6:** Distribution of outcome domains reported across the included studies (n = 13).

Included Study	Functional outcomes	Urological and sexual function	Clinical outcomes	Patient-reported outcomes (QoL and satisfaction)	Psychological outcomes	Postoperative recovery outcomes
Alberto Martini et al.,2024 [12]	N	Y	Y	N	N	N
Alessandro Uleri et al., 2025 [13]	N	Y	Y	N	N	N
Bogdan Adrian Buhas et al., 2025 [14]	N	Y	Y	N	Y	N
Guillaume Ploussard et al., 2020 [15]	N	N	Y	N	N	N
Razvan-George Rahota et al., 2022 [16]	N	Y	Y	N	N	N
Favil Singh et al., 2023 [17]	Y	Y	Y	Y	N	N
Malene Blumenau Pedersen et al., 2024 [18]	Y	Y	N	Y	Y	N
Santa Mina D et al., 2018 [19]	Y	Y	N	Y	Y	N
de Lira GHS et al., 2019 [20]	Y	N	N	N	N	N
Zhang Xinhong et al., 2024 [21]	Y	N	Y	N	Y	Y
Huang Yuhan et al., 2020 [22]	Y	N	Y	Y	Y	Y
Nie Ting et al.,2023 [23]	N	Y	Y	Y	Y	Y
Wang Fengying et al.,2025 [24]	N	Y	N	N	Y	N
Total (n)	6	9	9	5	7	3

Each row represents one included study, and each column represents a specific outcome domain. Y = Yes, outcome assessed; N = No, outcome not assessed.

The predominance of quasi-experimental designs and the wide variation in intervention duration reflect the early stage of evidence development in this field. Notably, the increasing number of publications since 2020 (Fig 2) suggests growing research interest in RP prehabilitation. However, the substantial methodological heterogeneity—particularly with respect to intervention timing and intensity—limits the comparability of findings across studies and underscores the need for more standardised research protocols in future investigations.

### Main intervention formats for prehabilitation in patients scheduled for RP

Based on delivery mechanism and clinical setting, the 13 included studies were categorised into three distinct formats: digital remote interventions (n = 4), non-digital remote interventions (n = 4), and in-person hospital-based guidance (n = 5). Fig 3 illustrates the distribution of these delivery models, and Table 4 presents an evidence map of intervention components by study.

Digital Remote Interventions Four studies [12–14,18] delivered prehabilitation via mobile applications. Three of these [12–14] employed the Betty.care application, which provided standardised and traceable interventions through preoperative assessment, remote monitoring, and structured educational modules encompassing exercise, PFMT, nutritional counselling, and postoperative follow-up. Notably, Buhas et al. [14] adopted a hybrid model combining Betty.care with supplementary in-person guidance. Pedersen et al. [18] utilised a custom-developed application integrating exercise prescription, psychological support, nutritional screening, and sexual health counselling.

Non-Digital Remote Interventions Four studies [15,17,19,23] delivered home-based prehabilitation via structured rehabilitation booklets combined with regular telephone follow-up. Booklets provided standardised guidance on exercise, nutritional management, and psychological support, while telephone follow-up served to address patient queries, promote adherence, and facilitate individualised exercise prescription adjustments.

In-Person Hospital-Based Guidance Five studies [16,20–22,24] delivered prehabilitation through face-to-face guidance during hospitalisation. Rahota et al. [16] conducted a one-day multidisciplinary workshop encompassing PFMT, limb exercises, and nutritional risk screening. The remaining four studies [20–22,24] combined bedside exercise instruction with nutritional assessment, psychological counselling, pain education, and relaxation training.

Notably, nearly two-thirds of the included studies (8/13) employed some form of remote or home-based delivery, reflecting a broader shift in perioperative care towards decentralised, patient-centred models. This trend is likely driven by the need for scalable interventions that minimise patient burden and accommodate resource constraints within clinical settings. However, the relative efficacy of remote delivery compared with in-person guidance remains unclear, as no included study directly compared these delivery approaches. Furthermore, while digital platforms offer advantages in terms of standardisation and traceability, they may inadvertently exclude older patients or those with limited digital literacy—subpopulations that constitute a substantial proportion of the PCa patient cohort. The hybrid model adopted by Buhas et al. [14], combining digital tools with in-person instruction, may represent a pragmatic compromise that preserves the respective benefits of both approaches while mitigating their individual limitations.

### Main intervention content of prehabilitation models in patients scheduled for RP

Table 4 summarises the frequency distribution of intervention components across included studies. Fig 4 presents the conceptual framework linking intervention components to reported outcomes.

#### Functional exercise.

All 13 included studies incorporated functional exercise as a core prehabilitation component.

(1) Pelvic floor muscle training (PFMT) was the most frequently implemented component, included in 11 studies [12–16,18–21,23,24]. Preoperative PFMT was initiated between one day and six weeks prior to surgery. Pedersen et al. [18] provided ultrasound-guided PFMT following initial clinical assessment, while de Lira et al. [20] employed electromyographic biofeedback to verify correct muscle activation technique.
(2) Aerobic training was incorporated in 10 studies [12–19,22–24], comprising activities such as brisk walking, stair climbing, and stationary cycling. Singh et al. [17] additionally incorporated rowing as an aerobic modality.
(3) Resistance training was included in four studies [17–19,22], utilising resistance bands and dumbbells. Singh et al. [17] prescribed leg press, chest press, and seated rowing exercises.
(4) Breathing exercises were included in six studies [14–16,21–23], with diaphragmatic breathing as the predominant technique; two studies [22,23] additionally incorporated effective coughing training to reduce postoperative pulmonary complications.
(5) Other exercise modalities included core stability training [17] and ankle pump exercises [22].

PFMT was the most consistently implemented component (11/13), reflecting its well-established role in post-RP urinary continence recovery. Aerobic training was similarly common (10/13), likely attributable to its established benefits for cardiorespiratory fitness and its ease of implementation in home-based settings. In contrast, resistance training was notably less prevalent (4/13). This discrepancy may reflect clinician concerns regarding exercise safety in patients with pelvic floor vulnerability, or alternatively, insufficient awareness of the evidence supporting resistance training for functional recovery in this population. Of particular importance, the substantial variation in training protocols—ranging from unsupervised home-based exercises to biofeedback-guided clinical sessions—introduces considerable heterogeneity that complicates the interpretation and synthesis of outcomes across studies.

#### Nutritional support.

Nine studies [12,14–16,18,21–24] incorporated nutritional interventions as a prehabilitation component. Seven studies [15,16,18,21–24] employed validated nutritional risk screening tools—specifically the Nutritional Risk Screening 2002 (NRS-2002)—to identify nutritionally at-risk patients preoperatively. Nutritional interventions encompassed oral nutritional supplementation, high-protein dietary guidance, enteral nutritional support, and lifestyle modification strategies including smoking and alcohol cessation, and increased fluid, lycopene, and selenium intake.

Although nutritional support was the second most frequently reported intervention component (9/13), a notable discrepancy exists between screening practice and outcome evaluation: while seven studies performed preoperative nutritional risk screening, only three [17–19] reported quantitative post-intervention nutritional outcomes (Table 5). This pattern suggests that nutritional prehabilitation is frequently implemented as a standard care component without rigorous evaluation of its clinical effectiveness. Given the well-established impact of malnutrition on surgical outcomes, future studies should prioritise objective nutritional assessment—incorporating body composition analysis or validated serum biomarkers—to better characterise the true value of nutritional prehabilitation in this population.

#### Other interventions.

(1) Psychological support was provided in five studies [18,21–24], encompassing validated anxiety and depression screening instruments (Hospital Anxiety and Depression Scale [HADS] [18,21]; State-Trait Anxiety Inventory and Centre for Epidemiological Studies Depression Scale [STAI/CES-D][19]; Self-Rating Anxiety Scale and Self-Rating Depression Scale [SAS/SDS][22]), mindfulness-based interventions, and relaxation training.
(2) Health education was delivered in three studies [13,14,24], providing structured patient education on catheter management, postoperative adverse effects, and pain management strategies.
(3) Sexual health counselling was provided in one study; Pedersen et al. [18] incorporated preoperative sexual health counselling as a discrete intervention component.
(4) Sleep intervention was included in one study; Wang et al. [24] incorporated sleep restriction therapy and the 4-7-8 breathing technique to address sleep quality prior to surgery.

Despite the recognised importance of psychosocial well-being in surgical recovery, psychological support was incorporated in only five studies, and sexual health counselling—a PCa-specific concern of particular clinical relevance—was notably absent from all but one included study [18]. This underrepresentation of psychosocial and sexual health components is clinically concerning, given that anxiety, depression, and erectile dysfunction are among the most distressing and persistent long-term consequences of RP for patients and their partners. Contributing factors may include intervention design complexity, the inherent sensitivity of psychosexual topics, or a historical research emphasis on more clinically tangible endpoints such as urinary continence recovery. Future prehabilitation protocols should address these domains more systematically, with potential integration into established psychosocial oncology services.

### Outcome measures in prehabilitation models for patients scheduled for RP

Six outcome domains were identified across the 13 included studies: urinary continence and sexual function; physical function and functional status; perioperative clinical outcomes; psychological status; health-related quality of life (HRQoL); and nutritional status. Table 5 details the outcome measures employed across studies, and Table 6 presents the frequency distribution across the six domains.

#### Urinary continence and sexual function.

Urinary continence was the most frequently assessed outcome domain, evaluated in 10 studies [12–14,16–20,22–24] using five distinct methodological approaches: pad use criteria [12–14], International Continence Society (ICS) criteria [23], 24-hour pad test [17–19], International Consultation on Incontinence Questionnaire–Urinary Incontinence Short Form (ICIQ-UI SF) [19,20], and urodynamic evaluation [24]. Sexual function was assessed in three studies [13,20,23] using the International Index of Erectile Function-5 (IIEF-5) [20,23] and the Sexual Life Quality Questionnaire (SLQQ-QOL [23]).

The primacy of urinary continence as an assessed outcome (10/13) underscores its clinical importance in post-RP recovery. However, the substantial methodological heterogeneity in continence assessment—ranging from simple binary outcomes (continent versus incontinent) to continuous quantitative measures (pad weight in grams; validated questionnaire scores)—severely limits cross-study comparability. The absence of a standardised core outcome set for continence assessment in the RP prehabilitation context represents a major barrier to evidence synthesis and meta-analytic investigation. Conversely, sexual function was evaluated in only three studies (3/13), representing a critical evidence gap given that erectile dysfunction is among the leading causes of long-term distress in RP survivors. This underrepresentation may reflect challenges in sexual health intervention design, patient and clinician discomfort with the topic, or a prevailing research emphasis on more readily measurable clinical endpoints.

#### Physical function and functional status.

Five studies [17–19,21,22] incorporated objective functional assessments. Four studies [18,19,21,22] employed the Six-Minute Walk Test (6MWT); two studies [18,19] used handgrip dynamometry; and Singh et al. [17] administered a comprehensive functional battery encompassing the chair stand test, 400-metre walk, and 6-metre fast and backward walk tests.

The 6MWT was the most commonly employed functional assessment tool (4/5 studies), consistent with its recommendation in prehabilitation guidelines as a validated measure of cardiorespiratory fitness and functional capacity. However, the diversity of supplementary assessments—spanning lower limb strength, upper limb strength, balance, and coordination—reflects an absence of consensus regarding the most clinically relevant functional domains for the RP patient population. A standardised multi-domain battery capturing both aerobic capacity and muscular strength is recommended to enable meaningful cross-study comparisons in future investigations.

#### Perioperative clinical outcomes.

Nine studies [12–17,21–23] reported perioperative clinical outcomes, encompassing three categories: postoperative recovery indicators (time to first ambulation [15,16,21,22]; time to first flatus [21–23]; time to catheter and drain removal [22,23]); hospitalisation-related indicators (length of hospital stay [15,16,21,22]; hospitalisation costs [15,22]; unplanned clinical visits [13,14,16]; readmission rates [12,15,16]); and surgical complications (Clavien-Dindo classification [12,13,16]; overall complication rates [21,22]).

Perioperative clinical outcomes were the second most frequently assessed domain (9/13), reflecting strong clinical interest in the safety and feasibility of prehabilitation. The adoption of the Clavien-Dindo classification in three studies [12,13,16] represents a positive methodological trend, as this standardised grading system facilitates cross-study comparison of complication severity. However, the broad range of additional indicators—spanning subjective patient-reported experiences to objective resource utilisation metrics—further highlights the absence of a harmonised outcome framework. Importantly, none of the included studies reported long-term oncological outcomes (e.g., biochemical recurrence-free survival, metastasis-free survival), representing a significant evidence gap, given that definitive cancer control constitutes the primary objective of RP.

#### Psychological status.

Five studies [18,19,21–23] assessed psychological outcomes, employing a range of validated instruments: HADS [18,21]; STAI/CES-D[19]; SAS/SDS [22]; and the Mishel Uncertainty in Illness Scale–Community version (MUIS-C) combined with the Fear of Progression Questionnaire–Short Form (FoP-Q-SF) [23].

Although psychological status was assessed in five studies, the utilisation of five distinct measurement instruments across these studies renders direct cross-study comparisons virtually impossible. This heterogeneity is particularly problematic given the high prevalence of anxiety and depression in PCa patients and their well-documented adverse impact on both surgical recovery and long-term quality of life. The incorporation of illness-specific measures (MUIS-C; FoP-Q-SF) in the Chinese studies [23] is noteworthy, as these instruments capture the unique psychological burden of cancer diagnosis, including illness uncertainty and fear of disease progression—dimensions not addressed by generic anxiety and depression scales. The absence of a widely validated, contextually appropriate instrument for psychological assessment in the RP prehabilitation setting represents a significant barrier to evidence synthesis in this domain.

#### Health-related quality of life.

Five studies [17–19,22,23] assessed HRQoL using a combination of generic cancer instruments and PCa-specific modules: EORTC QLQ-C30 [17]; SF-12 [18]; Functional Assessment of Cancer Therapy–General (FACT-G) [23]; Functional Assessment of Cancer Therapy–Prostate (FACT-P) [19]; and EORTC QLQ-PR55 [22].

The combined use of EORTC QLQ-C30 and the prostate cancer-specific QLQ-PR55 module [22] represents the internationally recommended approach for HRQoL assessment in this population, capturing both generic functional domains and disease-specific symptom burden. Nevertheless, the limited number of studies assessing HRQoL (5/13) and the heterogeneity of instruments employed suggest that patient-centred outcomes remain underprioritised in the current evidence base. Given that HRQoL represents the ultimate patient-centred endpoint in prehabilitation research, future studies should adopt validated, PCa-specific instruments to facilitate meaningful cross-study comparisons and meta-analytic synthesis.

#### Nutritional status.

Seven studies [15,16,18,21–24] employed the NRS-2002 for preoperative nutritional risk screening; however, only three studies [17–19] reported quantitative post-intervention nutritional outcomes, incorporating body composition analysis [17,18] or handgrip strength assessment [19].

The near-universal adoption of preoperative nutritional risk screening (7/9 studies incorporating nutritional interventions) contrasts markedly with the scarcity of objective post-intervention nutritional outcome assessment (3/9). This pattern suggests that while preoperative malnutrition risk is widely recognised, the actual impact of nutritional prehabilitation on patients’ nutritional status remains substantially under-evaluated. Given that body composition—particularly skeletal muscle mass—is an established predictor of surgical outcomes, the absence of standardised post-intervention nutritional assessment represents a significant evidence gap. Future investigations should incorporate validated body composition metrics, such as bioelectrical impedance analysis or computed tomography-derived muscle mass measurements, to rigorously establish the efficacy of nutritional prehabilitation in this population.

#### Other outcomes.

Four studies [14,21,23,24] reported additional outcome measures: patient satisfaction [14]; discharge readiness, assessed using the Readiness for Hospital Discharge Scale (RHDS) [21]; postoperative pain intensity, assessed using the Visual Analogue Scale (VAS) [23]; and sleep quality, assessed using the Insomnia Severity Index (ISI) [24].

Considered collectively, a clear and consistent pattern emerges across the 13 included studies: clinically tangible, readily measurable outcomes—urinary continence (10/13) and perioperative clinical indicators (9/13)—were substantially more frequently assessed than patient-centred psychosocial and functional outcomes, including psychological status (5/13), HRQoL (5/13), sexual function (3/13), and objective nutritional assessment (3/13). This pattern reflects a research culture that has historically prioritised short-term, clinically measurable endpoints over the multidimensional outcomes of greatest importance to patients. While this emphasis on surgical safety and objective clinical endpoints is understandable given the early stage of evidence development in this field, it represents a significant gap that future research must address. To advance the evidence base, future investigators should adopt a consensus-based core outcome set that incorporates both standardised clinical measures and validated patient-reported outcomes, thereby capturing the full spectrum of recovery following RP.

## Discussion

### Diversity of prehabilitation delivery formats and the emerging role of digital remote interventions

The 13 included studies employed three distinct delivery formats: in-person hospital-based guidance (n = 5) [16,20–22,24], non-digital remote interventions via structured booklets and telephone follow-up (n = 4) [15,17,19,23], and digital remote interventions via mobile applications (n = 4) [12–14,18]. This distribution reflects a progressive transition from traditional in-person guidance toward home-based delivery, driven by the need for scalable, patient-centred prehabilitation models that minimise burden on both patients and healthcare systems.

The growing adoption of digital prehabilitation in the RP population can be attributed to several converging factors. First, the COVID-19 pandemic served as a structural catalyst for telehealth adoption across healthcare systems, simultaneously creating the technical infrastructure and establishing patient familiarity necessary for remote intervention delivery. Second, digital platforms offer a scalable solution to resource constraints by enabling standardised, traceable interventions that require fewer in-person healthcare personnel. Third, patients increasingly express preference for home-based programmes that minimise travel burden, as evidenced by qualitative studies of prehabilitation experience [25]. Fourth, the proliferation of mobile health applications, coupled with advances in wearable biofeedback technology, has rendered remote delivery both technically feasible and commercially accessible at scale. In the included studies, digital platforms demonstrated particular promise in supporting integrated multicomponent delivery: the Betty.care application [12–14] provided preoperative assessment, remote monitoring, and structured educational modules, while Pedersen et al. [18] utilised a custom-developed application integrating exercise prescription, psychological support, nutritional screening, and sexual health counselling. A systematic review [26] confirmed the feasibility of technology-enabled cancer prehabilitation, although effectiveness was found to be contingent on intervention design quality and patient engagement mechanisms.

However, this trend toward digitalisation must be balanced against the risk of exacerbating existing health inequities. Patients with limited digital literacy or from socioeconomically disadvantaged backgrounds may be systematically excluded from purely digital programmes [27]—a concern of particular relevance given that PCa disproportionately affects older men, a demographic with lower rates of digital engagement. Each delivery format presents distinct advantages and contextual trade-offs. In-person hospital-based guidance affords the highest degree of clinical supervision, enabling real-time technique feedback and correction—particularly critical for PFMT, where biofeedback-guided instruction has been shown to improve correct muscle activation acquisition [22]. However, this format demands substantial healthcare personnel resources and imposes logistical travel burdens on patients. Non-digital remote interventions (structured booklets combined with telephone follow-up) represent a low-cost, accessible alternative requiring minimal technological infrastructure, and are particularly suitable for resource-limited settings or older populations with restricted digital literacy [15,17,19,23]; regular telephone contact additionally provides a human connection that may enhance patient engagement and adherence. Digital remote interventions offer the greatest potential for scalability and standardisation [12–14,18], with automated data capture enabling continuous monitoring and timely clinical adjustments. Notwithstanding these advantages, digital delivery faces challenges including the digital divide, the absence of direct supervision for exercise safety, and reliance on patient self-motivation for adherence [27].

The hybrid model adopted by Buhas et al. [14]—combining structured in-person instruction with digital follow-up—may represent an optimal pragmatic compromise, leveraging the strengths of both approaches while mitigating their respective limitations. In the Chinese healthcare context, preliminary digital platforms utilising WeChat mini-programmes have been developed [28,29], but these currently focus primarily on postoperative rehabilitation rather than preoperative optimisation. Future research should prioritise the development and evaluation of culturally adapted, preoperative digital platforms incorporating wearable biofeedback technology, with specific attention to accessibility for older and digitally underserved populations.

### Multimodal integration as a core strategy in prehabilitation content

All 13 included studies incorporated functional exercise, with PFMT representing the most consistently implemented component (11/13) [12–16,18–21,23,24], reflecting its well-established role in urinary continence recovery following RP. Aerobic training was incorporated in 10 studies [12–19,22,24], and resistance training in four [17–19,22], with functional improvements evidenced by 6MWT performance [18,19,21,22] and body composition analysis [17,18].

The clinical implications of multimodal versus unimodal prehabilitation warrant careful consideration, particularly in the context of RP where the multidimensional nature of postoperative recovery—encompassing urinary continence, erectile function, psychological well-being, and physical function—theoretically favours comprehensive, integrated approaches. Evidence from the broader surgical oncology literature provides important contextual support: meta-analyses have demonstrated that multimodal prehabilitation is associated with greater improvements in functional capacity and lower odds of postoperative complications compared with unimodal exercise programmes alone [30]. However, multimodal programmes simultaneously present significant adherence challenges. One study reported that while adherence was relatively high for exercise and nutritional components, it was substantially lower for psychological support, and that multimodal programmes demonstrated reduced overall adherence compared with unimodal approaches [31]. This adherence gradient suggests that programme complexity must be carefully calibrated against feasibility in individual patient contexts.

For the RP population specifically, a recent RCT of multimodal prehabilitation before robotic-assisted RP reported promising early improvements in quality of life and psychological outcomes [32]; however, no significant between-group differences were observed in physical function or erectile function during follow-up, suggesting that certain outcomes may require longer intervention periods, higher-intensity protocols, or more targeted approaches to yield detectable benefits. These null findings also highlight the importance of adequate statistical power and appropriate outcome timing in prehabilitation trials.

The heterogeneity observed across included studies is multifactorial and warrants structured analysis. First, intervention duration varied considerably from a single-day multidisciplinary workshop to six weeks of structured programming. Second, intervention frequency ranged from daily unsupervised home-based exercises to thrice-weekly supervised sessions. Third, delivery modality introduced variability in supervision intensity, feedback mechanisms, and patient engagement strategies. Fourth, patient characteristics including baseline functional status, comorbidity burden, cancer staging, and socioeconomic factors varied substantially across studies and settings, potentially acting as important effect modifiers. Fifth, outcome assessment timing ranged from immediate postoperative discharge to three months post-surgery, with the most common assessment time points being six weeks and three months, complicating comparisons of recovery trajectories across different phases of postoperative healing. Collectively, these sources of heterogeneity preclude definitive conclusions regarding the superiority of any single prehabilitation approach and underscore the urgent need for standardised research protocols.

### Heterogeneity of outcome measures and the need for a standardised evaluation framework

Considerable heterogeneity was observed across all assessed outcome domains. Urinary continence—the most frequently evaluated outcome (10/13) [12–14,16–20,23,24] was assessed using five distinct methodological approaches: binary pad use criteria [12–14], ICS continence standards [23], 24-hour pad weight testing [17–19], ICIQ-UI SF [19,20], and urodynamic evaluation [24]. This methodological diversity severely limits cross-study comparability and precludes quantitative synthesis through meta-analysis. For example, continence rates derived from binary pad use criteria (continent versus incontinent) differ substantially—both conceptually and quantitatively—from those derived from continuous pad weight measures (grams per 24 hours), rendering direct comparison between studies utilising these divergent methods methodologically problematic. Current clinical guidelines recommend a multimethod approach to continence assessment [33,34], underscoring the need for harmonised, consensus-based measurement protocols in future research.

This heterogeneity reflects a broader, well-recognised challenge within the prehabilitation literature. A scoping review of outcomes in randomised trials of surgical prehabilitation identified substantial variation in outcome reporting, prompting calls for the development of a consensus prehabilitation core outcome set [35]. Analogous efforts in colorectal surgery (the DiSCO initiative) [36] have employed Delphi consensus methodologies to achieve agreement on minimum outcome sets; adoption of equivalent approaches in the RP prehabilitation context would substantially improve the comparability of future research and facilitate evidence synthesis.

Sexual function, despite representing a primary post-RP concern and a major determinant of long-term patient well-being, was assessed in only three studies [13,20,23]. This critical evidence gap may reflect challenges in designing effective sexual health interventions, the inherent sensitivity of the topic for both patients and clinicians, or a historical research emphasis on more clinically tangible and readily measurable outcomes such as urinary continence recovery. Physical function assessment was anchored predominantly by the 6MWT (n = 4) [18,19,21,22], consistent with its endorsed status in prehabilitation guidelines. HRQoL was assessed using a combination of generic cancer instruments—including EORTC QLQ-C30 [17], SF-12 [18], FACT-G[23]and PCa-specific modules—FACT-P[19]and EORTC QLQ-PR55 [22]. The combined use of EORTC QLQ-C30 and the disease-specific QLQ-PR55 module represents the internationally recommended approach for comprehensive HRQoL assessment in this population [37], capturing both generic functional health domains and prostate cancer-specific symptom burden. Despite this, the diversity of instruments employed across the five studies assessing HRQoL limits direct comparability. Future research should systematically prioritise validated, PCa-specific instruments and align with emerging consensus-based core outcome sets to enable meaningful evidence synthesis.

## Barriers to implementation in different healthcare settings

The translation of prehabilitation into routine clinical practice faces substantial implementation barriers that vary considerably across healthcare systems and settings. A realist review identified common structural and individual-level barriers including patient transportation difficulties, insufficient social support, and inadequate information provision [38]; identified facilitators included programme individualisation, multimodal content, adaptation to local cultural and healthcare contexts, endorsement by clinical champions, and active multidisciplinary team involvement.

In the United Kingdom context, the three most clinically significant barriers have been identified as cost-effectiveness uncertainty, specialist workforce shortages, and the absence of national commissioning policy for prehabilitation services [39]. Importantly, a narrative review highlighted that prehabilitation programmes risk generating ‘intervention-based inequalities’, whereby patients with lower health literacy or from socioeconomically disadvantaged backgrounds are systematically less likely to engage with, adhere to, and benefit from structured prehabilitation [40]. This equity concern is particularly salient when considering the population-level implementation of digital and remote delivery models.

In the Chinese healthcare context, a recent nurse-led qualitative study [41] identified several context-specific implementation barriers, including: (1) suboptimal interprofessional collaboration and the absence of a dedicated specialist nurse role within multidisciplinary prehabilitation teams; (2) limited clinician training in prehabilitation delivery and the absence of standardised clinical pathways; (3) shortages of specialist human resources and insufficient hospital-level organisational support; and (4) constraints in medical equipment availability and information technology infrastructure. These barriers collectively impede the systematic, equitable delivery of prehabilitation at institutional scale. Nevertheless, substantial contextual opportunities exist within the Chinese healthcare environment: the near-universal adoption of WeChat as a communication platform, the rapid expansion of 5G digital infrastructure, and growing population-level acceptance of digital health services create a favourable technological landscape for remote prehabilitation delivery. Culturally adapted digital platforms that address health literacy needs, incorporate family and caregiver involvement—consistent with Chinese cultural values around illness and recovery—and integrate seamlessly with existing hospital electronic health record systems may offer a viable pathway to overcoming these implementation barriers at scale.

## Future research priorities

The evidence gaps identified in this scoping review indicate several specific and actionable research priorities. First, the development of standardised, consensus-based core outcome measures is urgently required. An international priority-setting study identified the three highest-ranked research priorities in prehabilitation as: (1) the effects of prehabilitation on surgical outcomes; (2) identification of patient subgroups most likely to derive benefit; and (3) determination of the optimal composition of prehabilitation programmes [42]. Defining core outcome measures was additionally identified as a distinct high-priority item, underscoring the critical importance of harmonised, internationally agreed outcome reporting standards. Second, well-powered RCTs incorporating patient-centred outcomes—including HRQoL, sexual function, and psychological well-being—are needed to establish the clinical efficacy of prehabilitation in the RP population with appropriate statistical rigour. Third, the optimal duration, timing, and component composition of multimodal prehabilitation programmes requires systematic investigation through factorial or component-wise trial designs. Fourth, digital and telehealth delivery models require rigorous evaluation with respect to clinical effectiveness, cost-effectiveness, and capacity to reach underserved patient populations, including older men and those with limited digital access. Fifth, implementation science research is required to evaluate the integration of prehabilitation into routine surgical care pathways, with a specific focus on identifying effective strategies to address barriers related to specialist workforce capacity, institutional infrastructure, reimbursement mechanisms, and health equity across diverse healthcare systems and cultural contexts.

Critically, it is essential to distinguish between feasibility and acceptability both of which this review broadly confirms for prehabilitation in the RP population—and clinical efficacy, which remains to be definitively established. The small sample sizes and predominance of quasi-experimental designs across the included studies necessitate that claims of effectiveness be made with appropriate caution. Rigorous trial designs with adequate statistical power, pre-specified standardised outcome measures, and sufficiently long follow-up periods are required to establish the true clinical value of prehabilitation for patients undergoing RP.

## Conclusion

This scoping review provides a comprehensive mapping of prehabilitation models for patients undergoing radical prostatectomy, synthesising evidence from 13 studies across three delivery formats and six outcome domains. The collective findings suggest that prehabilitation is feasible and acceptable for patients in the preoperative period; however, definitive evidence of clinical efficacy remains limited, attributable to the substantial heterogeneity of intervention designs and outcome measures, and the predominance of quasi-experimental studies with small sample sizes. Clinicians and researchers should therefore interpret the current evidence with appropriate caution, recognising the fundamental distinction between established feasibility and demonstrated efficacy.

Digital remote interventions are emerging as a promising and scalable delivery modality for prehabilitation, yet face unresolved challenges related to digital literacy, equitable access, and the digital divide. Multimodal prehabilitation may confer superior benefits compared with unimodal approaches, but requires careful programme design to maintain acceptable patient adherence. The substantial heterogeneity in outcome measures identified across included studies underscores the urgent need for an internationally agreed, consensus-based core outcome set for RP prehabilitation research. Future research should prioritise well-powered RCTs, systematic core outcome measure development, and implementation science investigations to advance the translation of prehabilitation from promising intervention to evidence-based standard of care.

## Limitations of study

Several limitations of this scoping review warrant acknowledgement. First, restriction to English- and Chinese-language publications may have introduced language bias, potentially excluding relevant studies published in other languages. Second, consistent with the methodological conventions of scoping reviews, formal risk-of-bias assessment was not conducted. Nevertheless, of the 13 included studies, six were RCTs and seven were quasi-experimental or prospective cohort designs; this predominance of non-randomised study designs should be explicitly considered when interpreting the strength of the evidence synthesised. Third, the substantial heterogeneity in intervention components, outcome measures, and outcome assessment timing across included studies precluded quantitative data synthesis or meta-analysis, limiting conclusions to a narrative and descriptive level. Fourth, the relatively small number of eligible studies (n = 13) reflects the nascent state of the evidence base in this specific clinical population, and inherently limits the breadth and generalisability of conclusions that can be drawn. Finally, the possibility of publication bias cannot be excluded, as studies reporting positive or statistically significant findings are more likely to be submitted and accepted for publication, potentially inflating the apparent effectiveness of prehabilitation interventions in this body of literature.

## Supporting information

S1 Checklist PRISMA-ScR checklist for this scoping review. (PDF)

S1 File Full search strategies. (DOCX)
