# Supplementary material for: Prehabilitation models in patients undergoing radical prostatectomy: A scoping review
Source: PLoS One. 2026 Aug 27;21(8):e0356745. doi: 10.1371/journal.pone.0356745 (PMC13521576; doi:10.1371/journal.pone.0356745)
Supplement: Supplementary file 2 [file pone.0356745.s002.docx]

**Full search strategies**

Table 1. **PubMed search strategy**

| Step | Search Strategy |
| --- | --- |
| #1 | "Prostatic Neoplasms"[Mesh] |
| #2 | "prostat* neoplasm*"[Title/Abstract] OR "prostat* cancer*"[Title/Abstract] OR "cancer of prostat*"[Title/Abstract] OR "cancer of the prostat*"[Title/Abstract] |
| #3 | #1 OR #2 |
| #4 | "Prostatectomy"[Mesh] |
| #5 | "prostatectom*"[Title/Abstract] OR "radical prostatectom*"[Title/Abstract] OR "robot* prostatectom*"[Title/Abstract] OR "laparoscop* prostatectom*"[Title/Abstract] |
| #6 | #4 OR #5 |
| #7 | "Preoperative Exercise"[Mesh] OR "Preoperative Care"[Mesh] OR "Exercise Therapy"[Mesh] OR "Physical Conditioning, Human"[Mesh] |
| #8 | "prehab*"[Title/Abstract] OR "preoperat* exercis*"[Title/Abstract] OR "preoperat* rehab*"[Title/Abstract] OR "preoperat* train*"[Title/Abstract] OR "preoperat* optim*"[Title/Abstract] OR "preoperat* interven*"[Title/Abstract] OR "preoperat* program*"[Title/Abstract] OR "exercise train*"[Title/Abstract] OR "physical train*"[Title/Abstract] OR "pelvic floor muscle train*"[Title/Abstract] OR "surgical prehab*"[Title/Abstract] OR "multimodal prehab*"[Title/Abstract] |
| #9 | #7 OR #8 |
| #10 | #3 AND #6 AND #9 |

**Web of science search strategy**

TS=((("prostat* neoplasm*" OR "prostat* cancer*" OR "cancer of prostat*" OR "cancer of the prostat*" OR "Prostatic Neoplasms" OR "Prostate Neoplasms") AND ("prostatectom*" OR "radical prostatectom*" OR "robot* prostatectom*" OR "laparoscop* prostatectom*" OR "Prostatectomy")) AND ("prehab*" OR "preoperat* exercis*" OR "preoperat* rehab*" OR "preoperat* train*" OR "preoperat* optim*" OR "preoperat* interven*" OR "preoperat* program*" OR "exercise train*" OR "physical train*" OR "pelvic floor muscle train*" OR "surgical prehab*" OR "multimodal prehab*" OR "Preoperative Exercise" OR "Preoperative Care" OR "Exercise Therapy" OR "Physical Conditioning" OR "Preoperative Rehabilitation" OR "Prehabilitation"))

**Embase search strategy**

('prostat* neoplasm*':ti,ab,kw OR 'prostat* cancer*':ti,ab,kw OR 'cancer of prostat*':ti,ab,kw OR 'cancer of the prostat*':ti,ab,kw OR 'prostate cancer'/exp) AND ('prostatectom*':ti,ab,kw OR 'radical prostatectom*':ti,ab,kw OR 'robot* prostatectom*':ti,ab,kw OR 'laparoscop* prostatectom*':ti,ab,kw OR 'prostatectomy'/exp) AND ('prehab*':ti,ab,kw OR 'preoperat* exercis*':ti,ab,kw OR 'preoperat* rehab*':ti,ab,kw OR 'preoperat* train*':ti,ab,kw OR 'preoperat* optim*':ti,ab,kw OR 'preoperat* interven*':ti,ab,kw OR 'preoperat* program*':ti,ab,kw OR 'exercise train*':ti,ab,kw OR 'physical train*':ti,ab,kw OR 'pelvic floor muscle train*':ti,ab,kw OR 'surgical prehab*':ti,ab,kw OR 'multimodal prehab*':ti,ab,kw OR 'prehabilitation'/exp)

**Cochrane Library search strategy**

("prostat* neoplasm*" OR "prostat* cancer*" OR "cancer of prostat*" OR "cancer of the prostat*") AND ("prostatectom*" OR "radical prostatectom*" OR "robot* prostatectom*" OR "laparoscop* prostatectom*") AND ("prehab*" OR "preoperat* exercis*" OR "preoperat* rehab*" OR "preoperat* train*" OR "preoperat* optim*" OR "preoperat* interven*" OR "preoperat* program*" OR "exercise train*" OR "physical train*" OR "pelvic floor muscle train*" OR "surgical prehab*" OR "multimodal prehab*")

**Scopus search strategy**

TITLE-ABS-KEY(("prostat* neoplasm*" OR "prostat* cancer*" OR "cancer of prostat*" OR "cancer of the prostat*") AND ("prostatectom*" OR "radical prostatectom*" OR "robot* prostatectom*" OR "laparoscop* prostatectom*") AND ("prehab*" OR "preoperat* exercis*" OR "preoperat* rehab*" OR "preoperat* train*" OR "preoperat* optim*" OR "preoperat* interven*" OR "preoperat* program*" OR "exercise train*" OR "physical train*" OR "pelvic floor muscle train*" OR "surgical prehab*" OR "multimodal prehab*"))

**China National Knowledge Infrastructure (CNKI) search strategy**

SU=(“prostate neoplasm” OR “prostate cancer” OR “prostate malignant*” OR “prostate* cancer”) AND SU=(“prostatectomy” OR “radical prostatectomy” OR “robotic prostatectomy” OR “laparoscopic prostatectomy”) AND SU=(“prehabilitation” OR “preoperative rehabilitation” OR “preoperative exercise” OR “preoperative training” OR “preoperative optimization” OR “preoperative intervention” OR “preoperative preparation” OR “exercise training” OR “physical training” OR “pelvic floor muscle training” OR “preoperative prehabilitation”)

**Wanfang Medical Database search strategy**

Topic:(“prostate neoplasm” OR “prostate cancer” OR “prostate malignant*” OR “prostate* cancer”) AND Topic:(“prostatectomy” OR “radical prostatectomy” OR “robotic prostatectomy” OR “laparoscopic prostatectomy”) AND Topic:(“prehabilitation” OR “preoperative rehabilitation” OR “preoperative exercise” OR “preoperative training” OR “preoperative optimization” OR “preoperative intervention” OR “preoperative preparation” OR “exercise training” OR “physical training” OR “pelvic floor muscle training” OR “preoperative prehabilitation”)

**China Biomedical Literature Database (SinoMed)search strategy**

(TI=“prostate neoplasm” OR TI=“prostate cancer” OR TI=“prostate malignant*” OR TI=“prostate* cancer”) AND (TI=“prostatectomy” OR TI=“radical prostatectomy” OR TI=“robotic prostatectomy” OR TI=“laparoscopic prostatectomy”) AND (TI=“prehabilitation” OR “preoperative rehabilitation” OR “preoperative exercise” OR “preoperative training” OR “preoperative optimization” OR “preoperative intervention” OR “preoperative preparation” OR “exercise training” OR “physical training” OR “pelvic floor muscle training” OR “preoperative prehabilitation”)

**VIP Chinese Journal Service Platform**

Su=(“prostate neoplasm” OR “prostate cancer” OR “prostate malignant*” OR “prostate* cancer”) AND Su=(“prostatectomy” OR “radical prostatectomy” OR “robotic prostatectomy” OR “laparoscopic prostatectomy”) AND Su=(“prehabilitation” OR “preoperative rehabilitation” OR “preoperative exercise” OR “preoperative training” OR “preoperative optimization” OR “preoperative intervention” OR “preoperative preparation” OR “exercise training” OR “physical training” OR “pelvic floor muscle training” OR “preoperative prehabilitation”)

**Notes:** All search terms were translated into Chinese when searches were conducted in the Chinese databases. The English translations are provided for transparency and reproducibility.
